# Modulation of BK channels contributes to activity-dependent increase of excitability through MTORC1 activity in CA1 pyramidal cells of mouse hippocampus

**DOI:** 10.3389/fncel.2014.00451

**Published:** 2015-01-13

**Authors:** Steven J. Springer, Brian J. Burkett, Laura A. Schrader

**Affiliations:** ^1^Neuroscience Program, Tulane UniversityNew Orleans, LA, USA; ^2^Department of Cell and Molecular Biology, Tulane UniversityNew Orleans, LA, USA

**Keywords:** whole cell patch clamp, afterhyperpolarization, theta burst stimulation, rapamycin, instantaneous frequency

## Abstract

Memory acquisition and synaptic plasticity are accompanied by changes in the intrinsic excitability of CA1 pyramidal neurons. These activity-dependent changes in excitability are mediated by modulation of intrinsic currents which alters the responsiveness of the cell to synaptic inputs. The afterhyperpolarization (AHP), a major contributor to the regulation of neuronal excitability, is reduced in animals that have acquired several types of hippocampus-dependent memory tasks and also following synaptic potentiation by high frequency stimulation. BK channels underlie the fast AHP and contribute to spike repolarization, and this AHP is reduced in animals that successfully acquired trace-eyeblink conditioning. This suggests that BK channel function is activity-dependent, but the mechanisms are unknown. In this study, we found that blockade of BK channels with paxilline (10 μM) decreased *I*_AHP_ amplitude and increased spike half-width and instantaneous frequency in response to a +100 pA depolarization. In addition, induction of long term potentiation (LTP) by theta burst stimulation (TBS) in CA1 pyramidal neurons reduced BK channel’s contribution to *I*_AHP_, spike repolarization, and instantaneous frequency. This result indicates that BK channel activity is decreased following synaptic potentiation. Interestingly, blockade of mammalian target of rapamycin (MTORC1) with rapamycin (400 nM) following synaptic potentiation restored BK channel function, suggesting a role for protein translation in signaling events which decreased postsynaptic BK channel activity following synaptic potentiation.

## Introduction

*N*-methyl-D-aspartate (NMDA) receptor-dependent long term potentiation (LTP) in area CA1 of the hippocampus has been linked to persistent changes in the intrinsic properties of the postsynaptic neurons and neuronal excitability (Daoudal and Debanne, 2003; Frick et al., 2004; Fan et al., 2005; Xu et al., 2005; Narayanan and Johnston, 2007; Jung et al., 2008). A change in intrinsic excitability modulates the cellular response to all synaptic inputs, not just the potentiated synapse or synapses, and thus can dramatically affect network function. The afterhyperpolarization (AHP) contributes to inter-spike interval and spike half-width and is a major determinant of intrinsic excitability. Several currents that contribute to the AHP are reduced following acquisition of hippocampus-dependent memory tasks and by the induction of synaptic plasticity (Disterhoft et al., 1996; Matthews et al., 2008, 2009; Matthews and Disterhoft, 2009; Oh et al., 2009; Cohen-Matsliah et al., 2010). The BK channels are voltage- and Ca^2+^-gated K^+^ channels that contribute to the fast AHP current (*I*_fAHP_; Lancaster and Nicoll, 1987; Storm, 1987) as well as action potential repolarization (Faber and Sah, 2003). Another family, the M-channels (Kv7/KCNQ), contributes to the medium AHP (*I*_mAHP_; Yue and Yaari, 2004, 2006; Hu et al., 2007), and this family of channels is important for synaptic integration and facilitation of LTP (Shah et al., 2011; Lee and Kwag, 2012; Petrovic et al., 2012).

BK channel activity in CA1 pyramidal cells is decreased following acquisition of trace-eyeblink conditioning (Matthews et al., 2008, 2009), and *in vivo* BK channel blockade in the dorsal hippocampus during training slowed task acquisition compared to controls (Matthews and Disterhoft, 2009) suggesting that they play a role in memory formation. These results suggest that the BK channels of the pyramidal cells of the hippocampus must be tightly regulated for optimal network function. Therefore, investigation of BK channel regulation by activity-dependent mechanisms is crucial to understand plasticity of intrinsic excitability.

Given the above information, we hypothesized that BK channel function is activity-dependent. We tested the sensitivity of CA1 pyramidal cells to paxilline (10 μM), a BK channel blocker, in control conditions and 2–3 h after LTP induction by theta burst stimulation (TBS). In control recordings, paxilline caused a significant decrease in *I*_AHP_ amplitude, and an increase in spike half-width and instantaneous firing frequency. Two-three hours after TBS-induced potentiation, paxilline application had no significant effect on *I*_AHP_ amplitude, spike half-width or instantaneous frequency. This result suggests that somatic BK channel activity is decreased in cells that undergo synaptic potentiation and that this decrease in *I*_AHP_ and BK channel contribution to spike firing contributes to the increase in postsynaptic excitability purported to play a permissive role in hippocampal memory formation and storage. We also investigated mammalian target of rapamycin (MTORC1) as a possible mechanism for regulation of BK channel currents post LTP-induction. We found that rapamycin application 30 min after TBS restored paxilline’s ability to increase spike repolarization and instantaneous frequency after TBS. This result suggests that protein translation by MTORC1 activation plays a role in activity-dependent regulation of BK channels following synaptic potentiation at the CA3-CA1 synapse.

## Methods

### Mice

Male 129 SVE mice from Charles River aged 3–7 weeks were used in all experiments. All experimental procedures were performed in accordance with the National Institutes of Health Guide for the Care and Use of Laboratory Animals and were approved by the Tulane University Institutional Animal Care and Use Committee.

### Electrophysiology

We performed voltage- and current-clamp recordings from CA1 pyramidal cells in acute hippocampus slices. Mice were anesthetized using isoflurane and rapidly decapitated. Hippocampal slices (350 μm) were cut in the transverse plane on a Vibratome. The cutting solution consisted of (in mM): 234 sucrose, 2.5 KCl, 1.25 NaH_2_PO_4_, 28 NaHCO_3_, 7 MgCl_2_, 0.5 CaCl_2_, 7 Dextrose, and 1 sodium ascorbate. Slices were incubated in a submerged chamber at 36°C for 30 min, then at room temperature thereafter until the time of recording. Recordings were done at 32°C. Individual neurons were visualized with an Olympus BX50WI fit using infrared illumination. The normal external recording solution (artificial cerebrospinal fluid, or ACSF) consisted of (in mM): 125 NaCl, 1.25 NaH_2_PO_4_ 2.5 KCl, 25 NaHCO_3_, 2 CaCl_2_, 1 MgCl_2_ and 10 dextrose, bubbled with 95% O2/5% CO_2_ at room temperature, pH 7.4. The internal pipette solution consists of (in mM): 120 Kgluconate, 20 KCl, 10 HEPES, 0.2 EGTA, 4 Mg_2_ATP, 0.3 Tris_2_GTP, 14 phosphocreatine, and 4 NaCl. All reagents used in intra- and extracellular solution were purchased from Sigma-Aldrich. Whole-cell patch recordings were made using the Axon MultiClamp 700B amplifier (Axon Instruments). Data were digitized at a rate of 10 KHz using Clampex 10.0 and analyzed using Clampfit 10.0 (Axon Instruments).

### Voltage clamp recordings

The *I*_AHP_ currents were evoked in voltage-clamp by holding the cell at −50 mV then depolarizing to +25 mV for 20 and 50 ms in order to elicit a maximum level of BK channel-mediated current. We determined the absolute *I*_AHP_ amplitude as the difference between the pre-pulse baseline and the peak of the tail current following the step offset. Peak *I*_AHP_ was compared between control and drug, either paxilline (10 μM, Sigma-Aldrich), iberiotoxin (100 nM, Sigma-Aldrich), rapamycin (400 nM, Tocris) or TBS using a paired *t*-test (control and drug).

### Current clamp recordings

Action potentials were evoked in each cell by a +100 pA, 1 s depolarization from a holding potential of −65 mV in control and treatment conditions. Since this depolarization evoked at least five spikes in all cells tested, we only analyzed the effect on the first five spikes for consistency, but the number of action potentials evoked by this current pulse in each condition is reported in Table 1. Changes in spike half-width from control traces were compared to those taken after the application of drugs or TBS.

**Table 1:** **Cell properties**.

	Spike amp (mV)	AHP amp (mV)	Threshold (mV)	Input resistance (MΩ)	Spike number	Sag (mV)	Animal #
Control	95.2 ± 1.7	−7.9 ± 0.8	−47.3 ± 0.9	200 ± 27.8	11.8 ± 1.1	1.9 ± 0.2	18
Paxilline	93.1 ± 1.9*	−6.9 ± 0.6	−50.3 ± 0.9****	212 ± 26.6	14.8 ± 1.2****	2.3 ± 0.2
Control	85.3 ± 7.5	−6.6 ± 1.4	−49.8 ± 1.6	197.6 ± 16.8	14.0 ± 3.2	2.1 ± 0.6	5
iberiotoxin	85.8 ± 4.7	−6.7 ± 1.4	−51.6 ± 1.2	239.0 ± 27.0	22.2 ± 2.8*	2.0 ± 0.8
Control	93.2 ± 1.3	−6.8 ± 0.6	−48.5 ± 1.1	183.8 ± 10.8	15 ± 2.1	1.5 ± 0.2	13
TBS	90.3 ± 1.3**	−5.7 ± 1.0	−50.0 ± 1.3	205.5 ± 15.2**	19 ± 2.1***	1.5 ± 0.2
TBS	95.0 ± 2.3	−8.2 ± 0.5	−49.0 ± 1.0	230.0 ± 32.4	15.2 ± 2.6	3.2 ± 0.5	9
TBS + Pax	93.5 ± 2.4	−8.2 ± 0.8	−49.8 ± 1.3	243.3 ± 38.0	15.6 ± 1.7	3.3 ± 0.6
Control	95.0 ± 1.5	−10.3 ± 1.0	−46.1 ± 1.3	180.0 ± 12.6	12.3 ± 2.1	2.4 ± 0.2	6
Rapamycin	91.5 ± 2.5	−7.4 ± 0.9***	−49.7 ± 1.1**	186.8 ± 11.6	15.5 ± 2.6**	2.4 ± 0.3
TBS+Pax	98.7 ± 2.1	−9.8 ± 1.0	−48.5 ± 1.0	227.3 ± 24	17.2 ± 3.5	3.4 ± 0.5	12
TBS+Pax + Rap	96.4 ± 1.8	−8.6 ± 1.4	−49.7 ± 1.8	233.3 ± 27.0	20.1 ± 3.9*	3.1 ± 0.5

The basic properties of cells from each group. The experimental group and its control are in one shaded group. The data are reported as mean ± SEM. The cell numbers are reported in the text. (*P < 0.05, **p < 0.01; ****p < 0.0001, all values are experimental compared to the control for that group, Students paired t-test).

**Figure 1:**
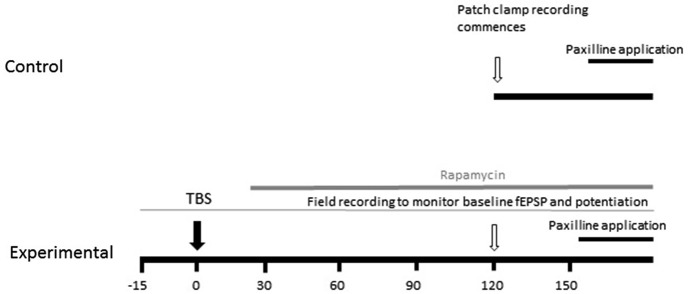
**Schematic diagram of experimental design**. The effect of TBS on paxilline-sensitive response was tested. In order to keep the duration from recording commencement to paxilline application consistent, TBS was applied 2 h before whole-cell patch clamp recording commenced. The field EPSP was monitored beginning 20 min prior to TBS to confirm potentiation.

Evoked excitatory postsynaptic potentials (EPSPs) were elicited by presynaptic stimulation via an isolated pulse stimulator (A-M Systems) and a bipolar stimulating electrode (Frederic Hauer) placed in the Schaeffer collaterals. Extracellular recordings were recorded from stratum radiatum of CA1 to monitor potentiation. To measure baseline properties, EPSPs were evoked every 10 s for a period of 5 min and averaged to analyze slope and amplitude. The experimental design is graphically illustrated in Figure 1. TBS consisted of three trains of 10 pulses of four 100 Hz pulses delivered at 5 Hz separated by an inter-train interval of 20 s. Subsequent EPSP measurements were made at 15, 30, 45, 60 and 90 min post-TBS. In order to analyze how the induction of synaptic plasticity affects somatic BK channel currents, we induced LTP by TBS, measured the increase in field excitatory postsynaptic potential (fEPSP) slope for 1.5 h, and then at 2–3 h patched an individual neuron in the slice which received TBS-LTP. Thus, the latency from commencement of recordings to paxilline application after TBS was similar to the control condition. We measured instantaneous frequency and spike half-width as described below, then applied paxilline to determine if the ability to produce an increase in spike half-width and instantaneous frequency was reduced following synaptic potentiation. We repeated the above experiment and applied rapamycin at 15–30 min after TBS, to determine the effect of MTORC1 blockade on the ability of paxilline to increase spike half-width and instantaneous frequency.

### Data analysis

All analyses were accomplished in Clampfit. Spike half-width was measured as the spike width at the half-maximal voltage. Specifically, the baseline threshold cursor was set to the cell’s baseline potential, i.e., the command potential of the cell, which was held at −65 mV. Half-width was calculated as the width of spike of the half-maximal voltage between the baseline and the peak of each spike. The event threshold cursor was set to ensure that the action potential crossed it in its rising and falling phase. The position of this cursor was not used in determining spike half-width. Spike threshold was measured as the point at least 2 standard deviations greater than the average of previous 0.01 ms, based on a sliding average. Spike height was measured from threshold to peak, and AHP amplitude was measured from threshold to the negative peak of the AHP. Sag was measured as the difference between the peak and steady state hyperpolarization produced by −60 pA current. Instantaneous frequency was determined as the inverse of interspike interval (1/ISI). Interspike interval was measured as the time between spike peaks. Spike half-width and instantaneous frequency in control and experimental conditions were compared using repeated measures ANOVA with repeated measures on spike number. The control and TBS spike-width was compared using an ordinary two-way ANOVA. In all cases, data are reported as mean ± SEM, *p* < 0.05 was considered significant.

## Results

### BK channels contribute to spike repolarization and instantaneous frequency in CA1 of mouse hippocampus

A total of 84 cells were recorded in this study. The basic properties of the recorded cells and the effect of drug application or TBS are reported in Table 1. The control and experimental groups are grouped together in shaded rows.

**Figure 2:**
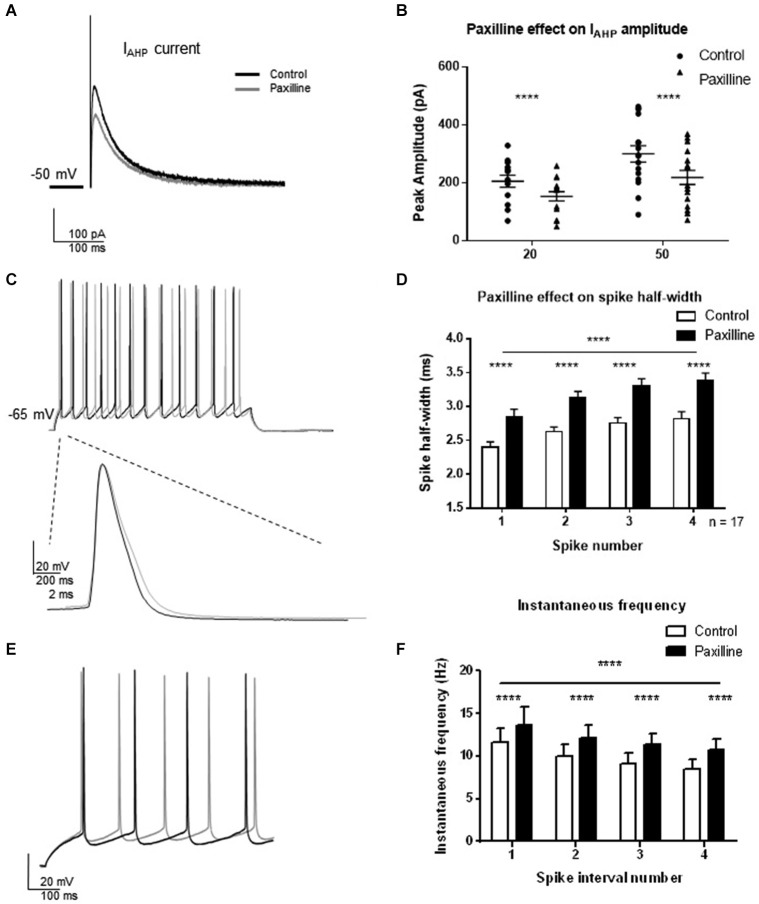
**Direct blockade of BK channels with paxilline application decreased *I*_AHP_ and increased spike half-width and instantaneous frequency. (A)** Paxilline application significantly reduced *I*_AHP_ amplitude compared to control at the 20 and 50 ms pulse lengths. Individual example showing reduced *I*_AHP_ tail current in response to paxilline application (gray) compared to control (black). **(B)** Summary bar graph showing *I*_AHP_ amplitude in control (filled circles) and paxilline (closed triangles). Paxilline had a significant effect on *I*_AHP_ amplitude at both the 20 and 50 ms depolarization (paired *t*-test, ****p < 0.0001). **(C)** Paxilline application resulted in a significant increase in spike half-width of the first four spikes in the spike train. Example of paxilline (gray) effect on the first spike compared to control (black). **(D)** Summary bar graph showing the effect of paxilline (black bars) across the first four spikes compared to control (white bars; (****p ≤ 0.0001). Significant spike broadening was observed across the train (****p < 0.0001). No significant interaction of paxilline application and spike number was observed. Data are reported as mean ± SEM. **(E)** Example of effect of paxilline application on spike instantaneous frequency in response to 100 pA depolarization. Paxilline application (gray) caused an increase in instantaneous frequency compared to control (black). **(F)** Summary bar graph showing the effect of paxilline (black bars) on the instantaneous frequency of the first five spikes compared to control (white bars). A decrease in instantaneous frequency was observed across interval number. Data are reported as mean ± SEM, (****p < 0.0001).

To assess the contribution of BK channels to *I*_AHP_ and spike firing properties of CA1 pyramidal cells, voltage- and current-clamp recordings from pyramidal cells from hippocampus slices were performed (Figure 2). In voltage-clamp, the *I*_AHP_ was evoked by direct current injection at the cell soma. Two pulse steps of different lengths (20 and 50 ms) to +25 mV from a holding potential of −50 mV were applied to evoke the *I*_AHP_, and changes in peak amplitude of the outward tail current were measured following termination of the step pulse in control and after addition of the BK channel blocker paxilline to the extracellular solution. At each pulse length, BK channel blockade with 10 μM paxilline significantly reduced the total *I*_AHP_ to approximately 70% of control (*t* = 5.8, df = 15, *p* < 0.0001, 20 ms pulse; *t* = 8.4, df = 15, *p* < 0.0001, 50 ms pulse; Figures 2A,B). In addition, paxilline application caused a significant increase in the spike half-width of the first four spikes of a +100 pA step depolarization (Figures 2C,D). Across the first four spikes, paxilline significantly increased spike half-width (*F*_(1,107)_ = 57.2; *p* < 0.0001; *n* = 17). A significant effect of spike number in the train was observed (*F*_(3,107)_ = 10.33; *p* < 0.0001), suggesting spike widening across the train. There was no significant interaction of paxilline and spike number (*F*_(3,107)_ = 0.18, *p* = 0.91). This suggests that BK channels play a role in spike repolarization, but they play no role in the spike widening across the train as previously reported in rat CA1 cells (Shao et al., 1999). The effect of BK channel blockade on instantaneous frequency during a depolarization was also determined. Paxilline application caused a significant increase in instantaneous firing frequency of the first five spikes (Figures 2E,F; *F*_(1,18)_ = 26.04; *p* < 0.0001), and there was a significant effect of interval number (*F*_(3,54)_ = 10.80; *p* < 0.0001), and no significant interaction (*F*_(3,54)_ = 0.73, *p* = 0.54, *n* = 17). These results suggest that currents provided by BK channels contribute to spike repolarization and instantaneous frequency. While other channels likely participate in these functions, we chose to focus on BK channels due to the consistent effect on spike repolarization and instantaneous frequency.

Previous reports using iberiotoxin to block BK channels showed that BK channels facilitate spike firing at high frequencies (>40 Hz), but had no effect at lower frequencies (Gu et al., 2007). Our results of an effect at lower instantaneous frequency in the present study seem inconsistent with this previous report. Several possibilities can explain this effect. Primarily, we used paxilline as the BK channel blocker whereas the previous study used iberiotoxin. While the BK molecular subunits that contribute to the instantaneous frequency are unknown, it has been reported that the β4 subunit contributes to differential sensitivity to iberiotoxin and paxilline (Meera et al., 2000). If these cells express the β4 subunit, different effects of iberiotoxin and paxilline may be observed.

**Figure 3:**
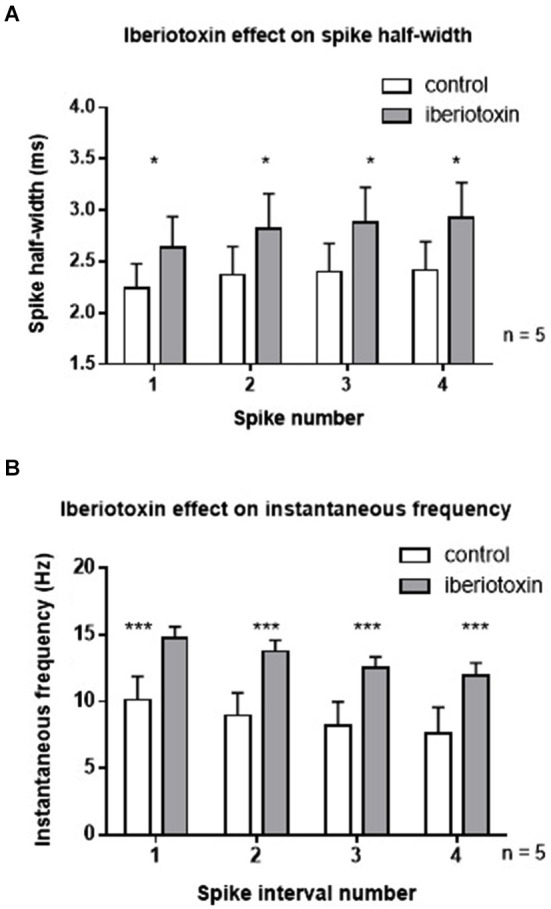
**Iberiotoxin (100 nM) mimicked the effects of paxilline on spiking activity. (A)** Iberiotoxin (gray bars) significantly affected the spike half-width across the first four spikes (*p < 0.05) compared to control (white bars). **(B)** Iberiotoxin application significantly increased instantaneous frequency (***p < 0.001). Data are reported as mean ± SEM.

In order to rule out differential effects of paxilline and iberiotoxin, the effect of iberiotoxin on spiking properties was determined (Figure 3). Similar to the results with paxilline, blockade of BK channels with iberiotoxin (100 nM) significantly increased the spike half-width of the first four spikes in the spike train (*F*_(1,32)_ = 4.5, *p* = 0.04; *n* = 5, Figure 3A). There was no significant effect of spike number (*F*_(3,32)_ = 0.23; *p* = 0.88) and no significant interaction (*F*_(3,32)_ = 0.01, *p* = 0.97). Furthermore, iberiotoxin application significantly increased instantaneous frequency of the first five spikes (*F*_(1,32)_ = 20.53; *p* < 0.0001; *n* = 5, Figure 3B). There was no significant effect of spike interval number (*F*_(3,32)_ = 1.39; *p* = 0.26), and no interaction (*F*_(3,32)_ = 0.01; *p* = 0.99). The similar results with iberiotoxin and paxilline in the present study suggest that BK channels regulate the spike repolarization and instantaneous frequency.

### Synaptic potentiation by TBS decreased BK channel activity 2–3 h post LTP induction

**Figure 4:**
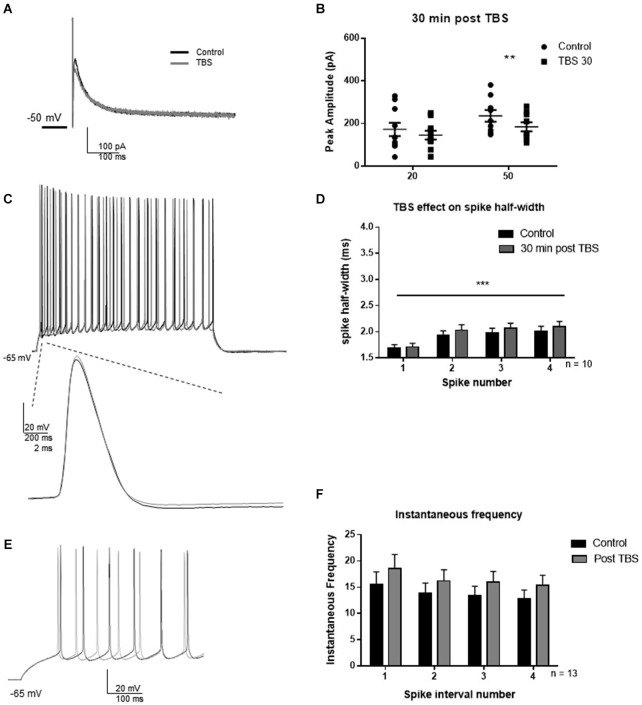
**TBS significantly reduced *I*_AHP_ amplitude, but not spike half-width and spike frequency 30 min after TBS. (A)** Example *I*_AHP_ tail current recorded after depolarization in control (black) and 30 min after TBS (gray). **(B)** Summary scatter plot of the peak amplitude of the *I*_AHP_ recorded at 20 and 50 ms depolarizations in control (closed circles) and 30 min after TBS (closed squares). TBS significantly reduced peak *I*_AHP_ at 50 ms (**p < 0.01). **(C)** Example of response to 100 pA depolarization in control (black) and after 30 min TBS (gray). Inset—example of a single action potential recorded in control (black) and 30 min after TBS (gray). **(D)** Summary bar graph of the half-width of the first four spikes in a spike train. TBS did not significantly affect spike half-width 30 min post-potentiation compared to control (white). Significant spike widening was observed across the first four spikes (***p < 0.0001). Data are reported as mean ± SEM. **(E)** Example of effect of TBS (gray) on the firing frequency in response to 100 pA depolarization compared to control (black). **(F)** Summary bar graph showing no effect of TBS on instantaneous firing frequency. Data are reported as mean ± SEM.

LTP was induced by TBS as described in methods to investigate whether synaptic potentiation influenced *I*_AHP_ and BK-dependent spiking characteristics (Figure 4). In an initial experiment, we assessed the effect of TBS on the *I*_AHP_ and firing properties. Thirty minutes following synaptic potentiation, *I*_AHP_ peak amplitude was reduced to 85 ± 12% of control in response to the 20 ms pulse (Figures 4A,B; *t* = 2.0; df = 9; *p* = 0.08), and 78 ± 9% of control in response to the 50 ms pulse (*t* = 4.0; df = 9; *p* = 0.004). Compared to control, TBS caused no significant effect on the half-width of the first four spikes 30 min post-TBS (Figures 4C,D; *F*_(1,64)_ = 1.27; *p* = 0.26, *n* = 9), significant spike widening across spikes occurred (*F*_(3,64)_ = 6.46; *p* = 0.0007) but no significant interaction (*F*_(3,64)_ = 0.07; *p* = 0.97). TBS had no significant effect on instantaneous frequency (Figures 4E,F; *F*_(1,96)_ = 3.16, *p* = 0.07), and there was no significant effect of interval number (*F*_(3,96)_ = 0.73; *p* = 0.54) and no interaction (*F*_(3,96)_ = 0.009; *p* = 0.99). These results suggest that TBS had no significant effect on the assessed measurements of intrinsic excitability at the 30 min post-TBS time point.

Previous results showed that regulation of the post-burst AHP by synaptic potentiation 3 h post-tetanus was protein translation-dependent (Cohen-Matsliah et al., 2010). We hypothesized that a greater decrease in BK channel currents may occur at a later time point after TBS. Therefore the time point of the study was extended to 2–3 h post-TBS, to determine if a greater decrease in BK channel activity occurred at this time point. In these experiments, TBS was induced 2–3 h before recordings commenced from the individual cell (see Figure 1). Therefore, the latency to paxilline application after commencement of recording was similar in control and after TBS.

**Figure 5:**
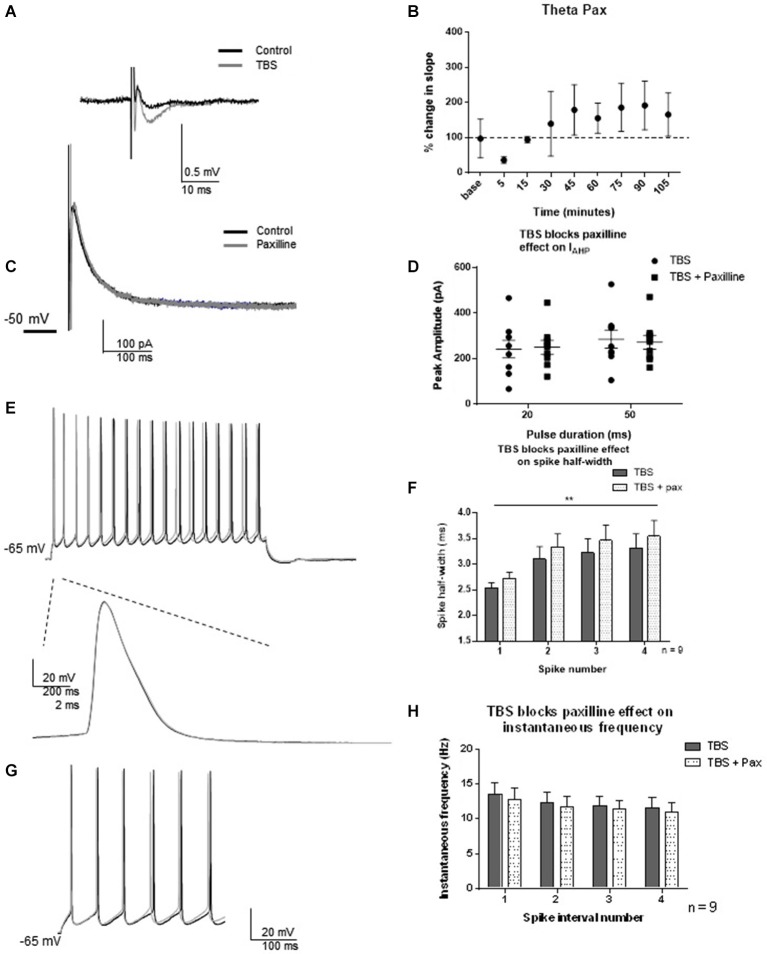
**TBS occluded the effect of blockade of BK channels with paxilline 2–3 h after TBS. (A)** Example of fEPSP in control and after TBS. **(B)** Bar graph showing the effect of TBS on fEPSP amplitude. TBS significantly increased fEPSP amplitude. **(C)** Example of peak *I*_AHP_ recorded 2–3 h following TBS in paxilline (gray) compared to control (black). **(D)** Summary scatter plot showing that paxilline (closed squares) had no effect on *I*_AHP_ amplitude 2–3 h following TBS compared to control (closed circles) **(E)** Example of voltage response to 100 pA recorded 2 h after TBS (black) and in the presence of paxilline (gray). Example of first spike in that train control (black) and paxilline (gray). **(F)** Summary bar graph showing the lack of effect of paxilline following TBS. As shown previously, spike half-width increased across the first four spikes (**p < 0.0001), and paxilline application (gray dippled) had no significant change compared to control after TBS (gray)**. (G)**. Example of instantaneous firing frequency recorded 2–3 h after TBS (black) and after TBS in paxilline (gray). Paxilline application had no significant effect on instantaneous frequency (dippled) after TBS compared to control (gray). **(H)**. Summary bar graph showing the results from the first five spikes. Data are reported as mean ± SEM.

As protein translation-dependent changes occur on a slower time scale than the post-translational modifications most commonly associated with channel regulation, we selected 2 h post-TBS as the time-point of analysis. LTP was induced by TBS, and fEPSP slopes were measured using field recordings in CA1 dendrites for 90 min to monitor potentiation. One hour post-TBS, TBS significantly increased the fEPSP slope (193 ± 64%) relative to control (*t* = 3.03; df = 6, *p* = 0.02, Figures 5A,B). Two hours post-TBS, whole-cell recordings from an individual neuron commenced and spike half-width and instantaneous frequencies were determined. Paxilline was then applied to determine if TBS modified the ability of paxilline to produce a decrease in *I*_AHP_ and changes in spiking properties. At this time point, synaptic potentiation by TBS blocked the ability of paxilline to decrease the *I*_AHP_ at both the 20 and 50 ms pulses (Figures 5C,D; 20 ms pulse, *t* = 0.77; df = 8; *p* = 0.47; 50 ms pulse *t* = 1.07; df = 8; *p* = 0.32; *n* = 9). No significant effect of paxilline on spike half-width was observed (Figures 5E,F; *F*_(1,64)_ = 1.64; *p* = 0.20, *n* = 9). As before, spike widening occurred and a significant effect of spike number was observed (*F*_(3,64)_ = 4.40; *p* = 0.007), but there was no significant interaction (*F*_(3,64)_ = 0.005; *p* = 0.99). In order to confirm an increase in spike-width 2–3 h after TBS, we compared the control recordings from this study to 2–3 h TBS. Indeed, TBS significantly increased spike half-width compared to control (*F*_(1,120)_ = 38.91; *p* < 0.0001). In addition, paxilline caused no further significant effect on instantaneous frequency after TBS (Figures 5G,H; *F*_(3,64)_ = 0.58; *p* = 0.63, *n* = 9). There was no significant effect of interval number (*F*_(1,64)_ = 0.35; *p* = 0.56) and no significant interaction (*F*_(3,64)_ = 0.002; *p* = 0.99). The results of the lack of effect of paxilline after TBS on *I*_AHP_ and instantaneous frequency in this study indicate a marked reduction in paxilline-sensitive BK channel activity that underlies spike repolarization and instantaneous frequency 2–3 h after synaptic potentiation.

### Blockade of MTORC1 after TBS restores BK channel function

**Figure 6:**
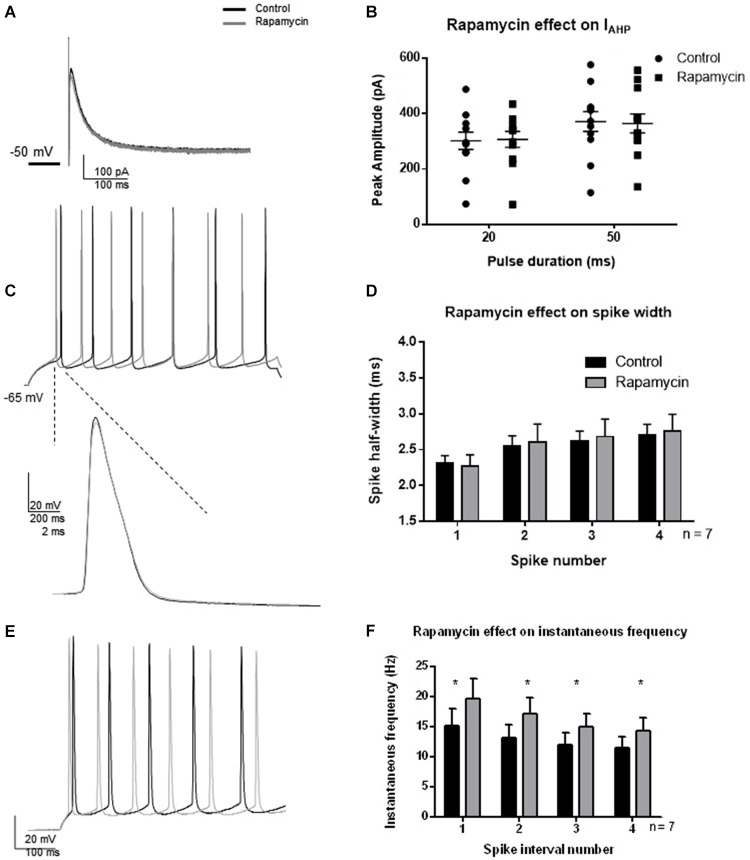
**Effect of rapamycin application (30 min) to block MTORC1 pathway on IAHP amplitude, spike half-width and instantaneous frequency. (A)** Example *I*_AHP_ tail current recorded after depolarization to +25 mV from −50 mV recorded in control (black) and application of rapamycin (gray). **(B)** Summary scatter plot of the peak amplitude of the *I*_AHP_ recorded at 20 and 50 ms depolarizations. Rapamycin application (closed squares) had no significant effect on the *I*_AHP_ current compared to control (closed circles). **(C)** Example of voltage response to a +100 pA depolarization in control (black) and rapamycin (gray). Inset—example of a single action potential recorded in control (black) and in rapamycin (gray). **(D)** Summary bar graph of the half-width of the first four spikes in a spike train. Rapamycin application (gray) had no significant effect on spike half width at each of the spikes investigated. **(E)** Example of instantaneous firing frequency recorded in rapamycin (gray) compared to control (black). **(F)** Summary bar graph showing instantaneous frequency of the first five spikes. Rapamycin significantly increased instantaneous frequency (*p < 0.05). Data are reported as mean ± SEM.

Since a strong activity-dependent regulation of BK channel activity was not observed 30 min following TBS and was temporally separated from synaptic potentiation by at least 1 h, we hypothesized that protein translation is necessary for the effect. We investigated whether protein translation by MTORC1 was necessary for the TBS-induced changes in BK function by blocking MTORC1 with rapamycin. Indeed, protein translation by MTORC1 mediates many functions in cells. Given the above results, we were specifically interested in activity-dependent regulation of BK channel activity. To ensure that rapamycin did not directly affect BK channel current or passive properties, we tested the effects of rapamycin in the absence of synaptic potentiation (Figure 6). Thirty minutes application of rapamycin significantly affected spike threshold and AHP amplitude in current clamp recordings (see Table 1). In addition, we found that rapamycin application (400 nM) had no significant effect on *I*_AHP_ current at 20 ms (Figures 6A,B; *t* = 0.48, df = 11; *p* = 0.64) or at 50 ms (*t* = 0.44, df = 11; *p* = 0.66). There was no significant effect of rapamycin on spike half-width (Figures 6C,D; *F*_(3,48)_ = 2.12; *p* = 0.11, *n* = 7), a significant effect of spike number (*F*_(1,48)_ = 0.065; *p* = 0.80) and no significant interaction (*F*_(3,48)_ = 0.038; *p* = 0.99). Rapamycin application did significantly affect instantaneous frequency (Figures 6E,F; *F*_(1,48)_ = 4.5; *p* = 0.04).

LTP was induced while extracellular recordings were performed and an individual neuron was patched 2–3 h after TBS. For this experiment, bath application of 400 nM rapamycin commenced 15–30 min post-TBS. This concentration of rapamycin was chosen based on previous results (Karpova et al., 2006). Rapamycin applied at this time point after TBS did not block potentiation of fEPSP amplitude, consistent with results which show that LTP is sensitive to MTORC1 blockade only during the induction phase (Sanna et al., 2002).

**Figure 7:**
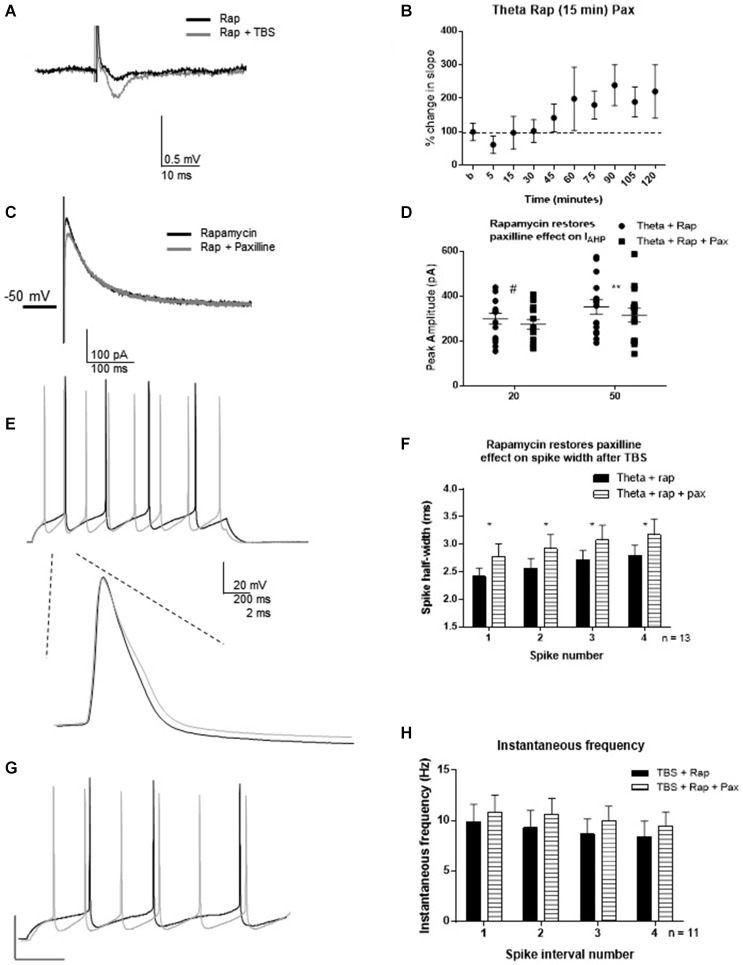
**Rapamycin application 30 min following synaptic potentiation restored the ability of paxilline to increase spike-width 2–3 h post-TBS. (A)** Example of fEPSP in control (Rapamycin) and after TBS (Rapamycin + TBS). **(B)** Bar graph showing the effect of TBS on fEPSP amplitude. TBS significantly increased fEPSP amplitude. **(C)** Example of *I*_AHP_ tail current recorded after TBS and rapamycin application (black) compared to addition of paxilline (gray). **(D)** Summary scatter plot showing the effect of paxilline application after TBS and rapamycin application (closed squares). Rapamycin significantly blocked the ability of TBS to reduce the paxilline sensitive *I*_AHP_ at 50 ms (**p < 0.01) and neared significance at 20 ms (^#^*p* = 0.052). **(E)** Example of the voltage response to 100 pA pulse recorded after TBS and rapamycin application (black) and after addition of paxilline. Inset—example of first spike in that train after TBS and rapamycin (black) and after addition of paxilline. **(F)** Summary bar graph showing that rapamycin after TBS restored the ability of paxilline to affect spike width after TBS. Paxilline application (horizontal lines) significantly affected spike width (*p < 0.05) after TBS and rapamycin application compared to TBS and rapamycin application (black). **(G)** Example of firing properties in response to 100 pA pulse from a cell recorded after TBS and rapamycin application (black) and after addition of paxilline (gray). **(H)** Summary bar graph showing that addition of paxilline (gray) did not significantly affect instantaneous frequency after TBS and rapamycin. Data are reported as mean ± SEM.

One hour post-TBS in rapamycin, TBS significantly increased the fEPSP slope (240 ± 55%) relative to control (Figures 7A,B). Therefore, rapamycin did not block LTP induction. Rapamycin incubation blocked the TBS-mediated decrease in *I*_AHP_ (Figures 7C,D), as subsequent paxilline application was able to produce further decrease in *I*_AHP_ amplitude (*n* = 15; 20 ms pulse: *t* = 2.12, df = 14; *p* = 0.052; 50 ms: *t* = 3.26; df = 14, *p* = 0.006). This suggests that TBS-induced MTORC1 activity is required for activity-dependent modulation of BK channels that underlie the *I*_AHP_ in addition to other functions. Further, rapamycin restored the ability of paxilline to affect the spike half-width. Paxilline significantly increased spike half-width (Figures 7E,F; *F*_(1,80)_ = 5.23; *p* = 0.02; *n* = 11), but there was no significant effect of spike number (*F*_(3,80)_ = 1.21; *p* = 0.31) and no significant interaction (*F*_(3,80)_ = 0.0006; *p* > 0.9999). On the other hand, paxilline had no significant effect on instantaneous frequency (Figures 7G,H; *F*_(1,80)_ = 1.100; *p* = 0.30), and there was no significant effect of interval number (*F*_(3,80)_ = 0.31; *p* = 0.820) and no interaction (*F*_(3,80)_ = 0.006; *p* = 0.99). The results of this experiment suggest involvement of the MTORC1 pathway in regulation of BK channels that play a role in spike repolarization in response to the induction of synaptic plasticity, as the ability of paxilline to produce an effect on spike repolarization and frequency was restored by rapamycin application 15–30 min post-TBS.

## Discussion

Here, we show that BK channel activity is reduced in an activity- and time-dependent manner, and MTORC1-dependent signaling is necessary for these effects, likely through protein translation. Previous studies have reported the effect of BK channel blockade on spike firing in CA1 pyramidal cells from rat hippocampus slices (Shao et al., 1999; Gu et al., 2007). Our results demonstrating the role of BK channels in spike repolarization and instantaneous frequency in CA1 pyramidal cells from mouse hippocampus slices are consistent with the earlier reports that showed that BK channels contribute to spike repolarization, however, our studies did not demonstrate participation in the spike widening across the depolarization as previously reported (Shao et al., 1999).

Blockade of BK channels can either increase (Matthews et al., 2008) or decrease firing rate. The decrease in firing rate is seen particularly during high excitability conditions such as epileptiform seizures (Jin et al., 2000; Brenner et al., 2005; Du et al., 2005). Specifically relevant to this study, a previous report showed that blockade of BK channels in CA1 pyramidal cells reduced early spike discharge frequency at high frequency firing rates (>40 Hz) and had no effect at lower frequencies (Gu et al., 2007). While our results seem inconsistent with this report, we did not study high frequency firing rates (>40 Hz). We do show, however, that blockade of BK channels increases firing frequency as was previously shown (Matthews and Disterhoft, 2009). While we did not experimentally test the presence of the β4 subunit, we did test the effect of both paxilline and iberiotoxin, and no differences between paxilline and iberiotoxin were observed. This suggests that interaction with β4 is not a significant contributor to the channels important for spike repolarization or instantaneous frequency. Other subunits, however, may be involved in regulation of spike width. The low frequencies tested in our study (<20 Hz) were lower than those tested in the previous study (Gu et al., 2007). Therefore, BK channels may mediate opposing and bidirectional effects at low (<20 Hz) and high (>40 Hz) frequencies in CA1 pyramidal cells. Further studies are needed to determine the specific role of BK channels in this possibility.

We showed that the induction of synaptic plasticity by TBS caused a reduction in the BK channel contribution to spike repolarization at a late time point hours after plasticity induction. This result shows activity-dependent regulation of the BK channels that participate in spike repolarization and supports previous findings that show that acquisition of a hippocampus-dependent memory task caused decreased *I*_fAHP_, increased spike half-width, and decreased responsiveness to direct BK channel blockade by paxilline (Matthews and Disterhoft, 2009).

The demonstration that somatic CA1 BK channel activity is reduced following synaptic potentiation is an important connection between the induction of synaptic plasticity and the regulation of somatic excitability. A decrease in somatic BK channel activity could affect a neuron’s intrinsic excitability in several ways. Activity-dependent regulation of BK channels may be a homeostatic mechanism to prevent further plasticity induced by high frequency firing, but may enhance plasticity at low frequencies (i.e., LTD) and ensure a homeostatic decrease in synaptic efficacy. Also, as BK channels limit calcium entry which occurs during the falling phase of spike firing, the increased calcium entry from spikes early in a spike train might lead to a shift toward increased calcium-dependent intracellular signaling following plasticity induction. However, the role of this reduction in BK channel activity still remains in question in the larger picture of the role of alterations in somatic excitability accompanying synaptic changes.

### A role for MTORC1 in LTP-mediated reduction of BK channel-mediated spiking effects

The delayed response of BK channel activity reduction after TBS was consistent with a role of protein translation in activity-dependent BK channel regulation. Therefore we investigated the role of the MTORC1 pathway in activity-dependent modulation of BK channel-mediated spike effects. In our experiments, rapamycin application alone affected only the threshold. It is important to note, however that MTORC1 has been shown to regulate Kv1.1 channel expression by activity (Raab-Graham et al., 2006). Since it was important that synaptic potentiation was obtained, we began rapamycin incubation 15–30 min following TBS. While we did not investigate MTORC1 activation at this time point, previous studies suggest that MTORC1 is activated after strong stimulation that induces long-lasting LTP (Tsokas et al., 2005). We demonstrated that incubation of rapamycin 15–30 min following TBS restored the ability of paxilline application to mediate its effects on spiking properties. This result indicates that active BK channels are present following TBS and MTORC1 blockade by rapamycin, and that when MTORC1 activity is blocked near the induction of synaptic plasticity, a pool of active BK channels remains available, based on the restored effect of paxilline. MTORC1 is an important contributor to the regulation of protein translation which contributes to late-phase LTP (Kelleher et al., 2004). LTP induction by HFS increases MTORC1 activation both at the potentiated synapse and at the cell body (Man et al., 2003). We show in our study that MTORC1 activity does contribute to regulation of BK channel activity that underlies instantaneous frequency following synaptic potentiation. Overall these studies suggest a possible mechanism to the complex contribution of BK channels in memory formation and suggest a role for BK channels in homeostatic mechanisms of excitability modulation.

Taken together, these results pose a larger question. Do changes in BK channel currents occur as a consequence of memory-related synaptic potentiation that help stabilize synaptic changes, or do they occur prior to memory acquisition to support activity-dependent changes? Our results demonstrating the delayed reduction in BK channel activity after TBS suggest that the changes in BK channel activity act to maintain changes, rather than play a role in the induction of plasticity itself. Further studies are necessary, however, to determine the specific role and time point of BK channels in memory acquisition.

## Conflict of interest statement

The authors declare that the research was conducted in the absence of any commercial or financial relationships that could be construed as a potential conflict of interest.
